# State-Dependent Blockade of Dorsal Root Ganglion Voltage-Gated Na^+^ Channels by Anethole

**DOI:** 10.3390/ijms25021034

**Published:** 2024-01-14

**Authors:** Luiz Moreira-Junior, Jose Henrique Leal-Cardoso, Antonio Carlos Cassola, Joao Luis Carvalho-de-Souza

**Affiliations:** 1 Department of Anesthesiology, University of Arizona, Tucson, AZ 85724, USA; luizmoreira@arizona.edu; 2 Superior Institute of Biomedical Sciences, State University of Ceará, Campus of Itaperi, Fortaleza 607402, CE, Brazil; 3 Department of Physiology and Biophysics, Biomedical Sciences Institute, University of Sao Paulo, São Paulo 05508, SP, Brazil

**Keywords:** anethole, sodium channel, state-dependent blockade, dorsal root ganglion, patch clamp

## Abstract

Anethole is a phenolic compound synthesized by many aromatic plants. Anethole is a substance that humans can safely consume and has been studied for years as a biologically active molecule to treat a variety of conditions, including nerve damage, gastritis, inflammation, and nociception. Anethole is thought to carry out its biological activities through direct interaction with ion channels. Anethole is beneficial for neurodegenerative Alzheimer’s and Parkinson’s diseases. Nevertheless, nothing has been investigated regarding the effects of anethole on voltage-gated Na+ channels (VGSCs), which are major players in neuronal function. We used cultured dorsal root ganglion neurons from neonatal rats as a source of natively expressed VGSCs for electrophysiological studies using the whole-cell patch-clamp technique. Our data show that anethole interacts directly with VGSCs. Anethole quickly blocks and unblocks (when removed) voltage-activated Na+ currents in this preparation in a fully reversible manner. Anethole’s binding affinity to these channels increases when the inactive states of these channels are populated, similar to lidocaine’s effect on the same channels. Our data show that anethole inhibits neuronal activity by blocking VGSCs in a state-dependent manner. These findings relate to the putative anesthetic activity attributable to anethole, in addition to its potential benefit in neurodegenerative diseases.

## 1. Introduction

**Figure 1:**
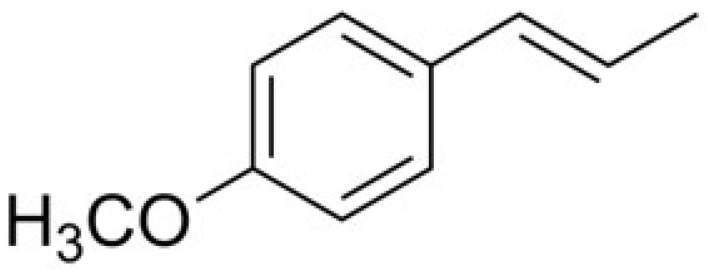
Chemical structure of anethole.

Anethole (ANE, IUPAC name 1-methoxy-4-[(E)-prop-1-enyl]benzene, Figure 1) is an anisole derivative compound produced by plants such as anise (*Pimpinella anisum*), fennel (*Foeniculum vulgare*), star anise (*Illicium verum*), and tarragon (*Artemisia dracunculus*). ANE is the major component of the essential oils of anise and fennel, accounting for 96.8% and 83.43% of their oil compositions, respectively [1]. In these plants, essential oils represent 1.5–6% of their weight [2]. In plant biology, phenylpropanoids like ANE are key to plants’ defense responses against other forms of life, plant-to-plant communication, and attracting pollinators [3]. To humans, these essential oils first represented a natural source of flavoring substances as well as antimicrobials for food preservation and wound healing. Next, essential oils represented a source of biologically active molecules that possess high therapeutic indices. Purified constituents of essential oils have been extensively studied as modulators of a vast diversity of biological processes. For example, ANE has been demonstrated to have anti-inflammatory, anticancer, and antidiabetic properties, and to be neuroprotective against ischemic brain damage [4]. In animal models and isolated animal organs, ANE has been successfully tested as an antihypernociceptive and anti-edematogenic [5], an antispasmodic and a myorelaxant in smooth and skeletal muscle preparations [6,7,8], an antinociceptive, a nerve excitability blocker [9], a gastroprotector [10], an antihypertensive [11] and an antithrombotic [12]. Although these effects are undeniably attributable to ANE, they may also be due to ANE hepatic metabolites that are pharmacologically active [13]. Interestingly, ANE has beneficial effects in Alzheimer’s and Parkinson’s diseases, and these effects are thought to be due to the inhibition of cholinesterase induced by ANE [14].

The biological activities of ANE may be carried out through direct interaction with ion channels, and studies in this direction are scarce. ANE is a selective agonist of mouse and human TRPA1, a TRP channel implicated in sensory transduction in peripheral neurons that is also activated by mustard oil [15,16]. In this role, ANE may produce algesia. In a study conducted in *Helix aspersa* neurons, ANE was suggested to block voltage-gated Ca^2+^ channels and Ca^2+^-activated K^+^ channels [17,18,19]. In sperm cells, ANE decreased the CATSPER current to impair sperm function [20]. Elegant studies using isolated rat aorta as a tissue model suggested ANE to be a voltage-gated Ca^2+^ channel activator, but further investigation is necessary to confirm this role [21].

Despite the suggestive and interesting effects of ANE on ion channels, this molecule is poorly explored as such. We have identified this knowledge gap, and closing it is key to understanding ANE as a molecule that is biologically active on excitable cells, especially neuronal cells. Moreover, since ANE exhibits beneficial effects in neurodegenerative diseases like Alzheimer’s and Parkinson’s diseases, further studies about the molecule’s effects on neuronal excitability are necessary. Here, we show novel data that start to close this knowledge gap. We studied the effects of ANE on voltage-gated Na^+^ channels (VGSCs) expressed in sensory neurons from rats. These neurons represent a model to study VGSCs natively expressed in their physiological environment. ANE blocks voltage-activated Na^+^ currents (I_Na+_) passing through these VGSCs in a dose-dependent manner and in a fully reversible fashion. ANE changes the voltage dependence of the activation and inactivation processes, and it enhances the time-dependent inactivation of I_Na+._ Our data indicate that ANE binds to the resting states of VGSCs as well as to their inactive states with enhanced affinity.

## 2. Results

### 2.1. Anethole Reversibly Inhibited Total Sodium Currents (I_Na_+) in DRG Neurons

**Figure 2:**
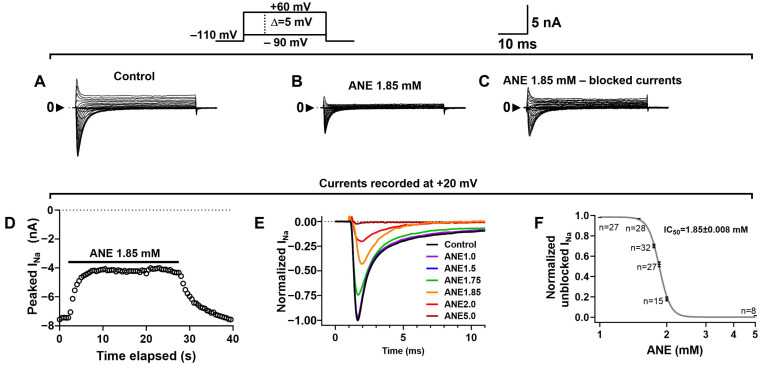
Anethole blocks total sodium current (I_Na+_) in a concentration-dependent and fully reversible manner. (**A**) Representative I_Na+_ recorded in the absence of ANE (control) at voltages ranging from −90 to +60 mV, as indicated. (**B**). Representative traces showing I_Na+_ recordings in the presence of 1.85 mM ANE. (**C**) Representative I_Na+_ that were blocked by 1.85 mM ANE. (**D**) A typical time course of I_Na+_ blockade by ANE evidencing a quick blockade of the currents that are fully reversible. (**E**) Typical I_Na+_ recordings in the presence of several ANE concentrations in different cells, as indicated by the color code. (**F**) Concentration-dependent blockade of I_Na+_. The currents were blocked by ANE with an IC50 of 1.85 mM ± 0.008 mM (n varies from 8 to 32 neurons) and a Hill slope coefficient of 16.69 ± 1.580. All recordings in this figure were obtained with voltage steps from a holding potential of −110 mV. The effects of ANE on the holding currents are negligible.

We studied I_Na+_ recorded from rat dorsal root ganglia (DRG) neurons using voltage-clamp methods. All other currents from these cells were eliminated by blockers, as detailed in the methods section. I_Na+_ recordings were obtained using voltage steps from a holding potential of −110 mV, and ANE shows no effects on the holding currents. We show that ANE (Figure 1) inhibited I_Na+_ at all membrane potentials (Figure 2A–C). The blockade is fully reversible and concentration-dependent (Figure 2D–F). Typical I_Na+_ traces in the absence and presence of different concentrations of ANE, each in a different neuron, are shown. ANE blocks I_Na+_ with an IC50 of 1.85 ± 0.008 mM and with a Hill slope coefficient of 16.69 ± 1.580 (Figure 2F), per our fitting procedure using Hill’s equation with a variable slope (Equation (1), see methods). In Figure 2, ‘n’ indicates the number of neurons evaluated with each concentration of ANE. Additional tests with ethanol only show no significant effects of ethanol on the I_Na+_ studied here (Supplementary Figure S1).

### 2.2. Anethole Has a Frequency-Dependent Inhibition Effect on I_Na+_

**Figure 3:**
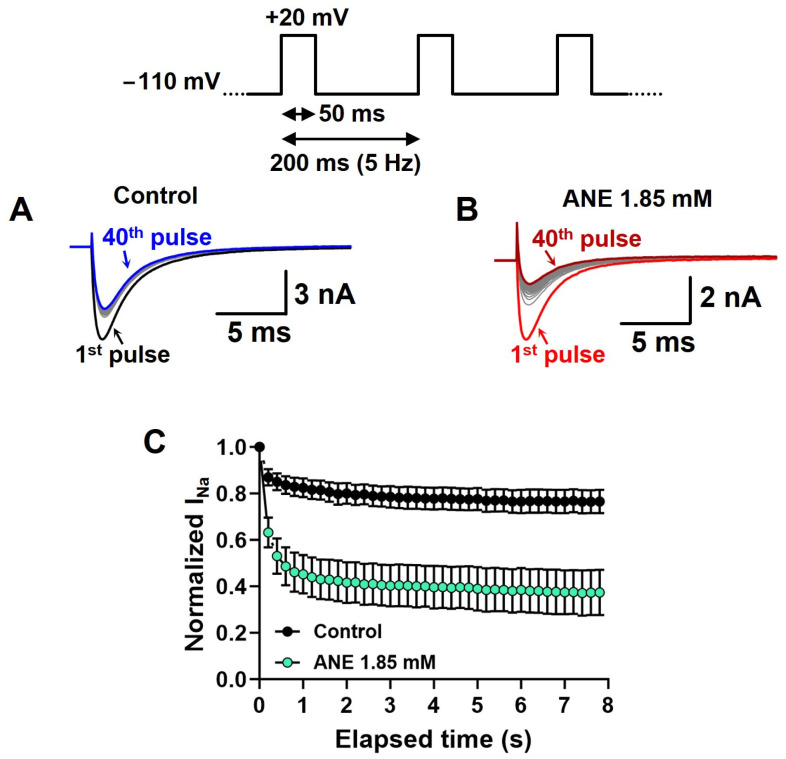
I_Na+_ blockade induced by ANE is enhanced at high-frequency depolarizations. (**A**,**B**) Typical I_Na+_ traces showing intensified blockade induced by ANE over 40 successive depolarizations at 5 Hz. (**C**) Averaged (symbols) ± SEM (bars) effect of ANE on I_Na+_ peaks after normalization by first pulse peak amplitude in each condition. All recordings in this figure were obtained with voltage steps from a holding potential of −110 mV. The effects of ANE on the holding currents are negligible. The two conditions (control vs. ANE) are statistically different when tested using a 2-way ANOVA (n = 8 neurons, *p* < 0.0001).

We assessed the frequency-dependent inhibition of I_Na+_ by ANE using a time series of depolarizations at 5 Hz with 50 ms periods at +20 mV from a holding potential of −110 mV. For these experiments, we did not use the P/−4 protocol to subtract leak currents and uncompensated transients (see methods) (Figure 3A,B). These experiments evaluated the inability of VGSCs to recover from inactivation in between two consecutive voltage pulses. Our data show normalized I_Na+_ peaks during the depolarization series in the absence and in the presence of ANE at 1.85 mM (Figure 3C). Typically, the I_Na+_ blockade induced by ANE was significantly increased during the 5 Hz series from 48% in the first pulse to 71% I_Na+_ blockade in the 40th depolarizing pulse (n = 7, paired *t* test, *p* = 0.0002, Figure 3C).

### 2.3. Anethole Shifts the Voltage Dependence of Na^+^ Conductance Activation

**Figure 4:**
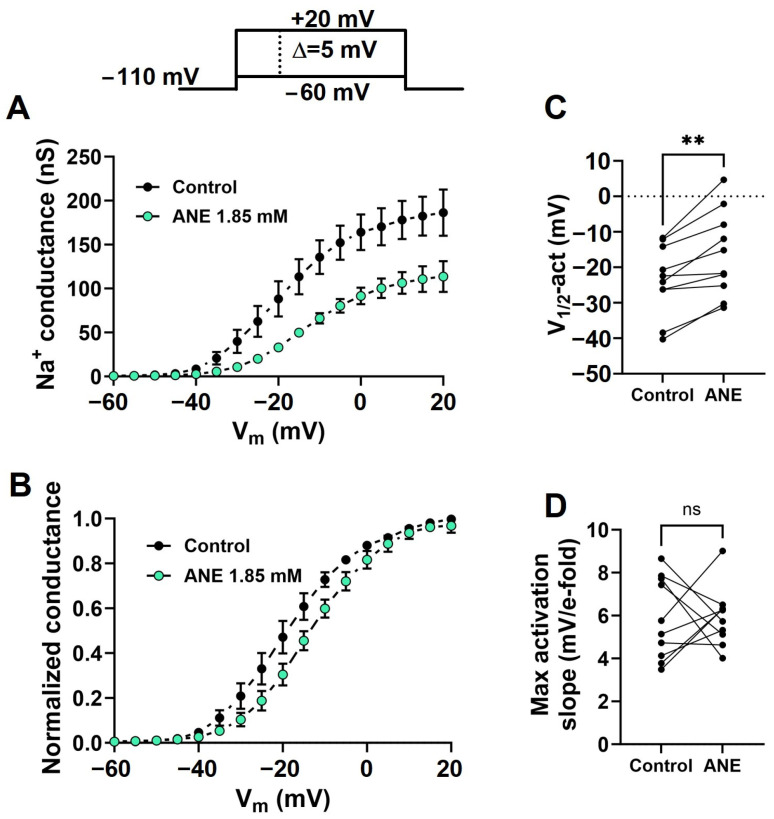
Anethole changes the voltage dependence of the I_Na+_ activation process. (**A**) Conductance-voltage (G-V) curves in the absence (control) and in the presence of 1.85 mM ANE. (**B**) Normalized and averaged G-V curves in the absence (control) and in the presence of ANE at a 1.85 mM concentration. Data from individual cells were plotted as individual G-V curves and fitted using Boltzmann’s equation for calculating the G-V curves’ midpoint (V_1/2-_act, **C**) and Max activation slope (**D**) parameters. All recordings in this figure were obtained with voltage steps from a holding potential of −110 mV. The effects of ANE on the holding currents are negligible. ** denotes paired *t* test, *p* < 0.001; ns, not significant (n = 10 neurons).

The current–voltage relationships were recorded using a series of depolarizing pulses ranging from −60 mV to +20 mV in steps of 5 mV, with 5 s intervals, from a holding potential of −110 mV. Using Ohm’s law (Equation (2), see methods), we transformed each I_Na+_ peak value into Na^+^ conductance and used that as a measure of the maximal conductance at each depolarizing voltage to build G-V curves in the absence and presence of ANE (Figure 4A). We fitted individual G-V curves with Boltzmann functions (see methods) to extract the maximal conductance, voltage dependence (V_1/2_-act), and voltage sensitivity (Max activation slope) of the activation process. We plotted average normalized G-V curves to highlight the shift in the voltage dependence induced by ANE (Figure 4B). From our analysis with fitting data using Boltzmann’s formalism (Equation (3), see methods), we learned that ANE at 1.85 mM effectively decreases the maximal Na^+^ conductance from 169.8 ± 20.55 to 111.1 ± 16.23 nS (n = 10, paired *t* test, *p* = 0.0004). Further, ANE shifts the voltage dependence by +7.3 ± 1.55 mV (n = 10, paired *t* test, *p* = 0.0011, Figure 4C). However, ANE does not significantly change the voltage sensitivity of the activation process (n = 10, paired *t* test, *p* = 0.9522, Figure 4D).

### 2.4. Anethole Does Not Interact Specifically with Voltage-Gated Na^+^ Channels in Pre-Open States

**Figure 5:**
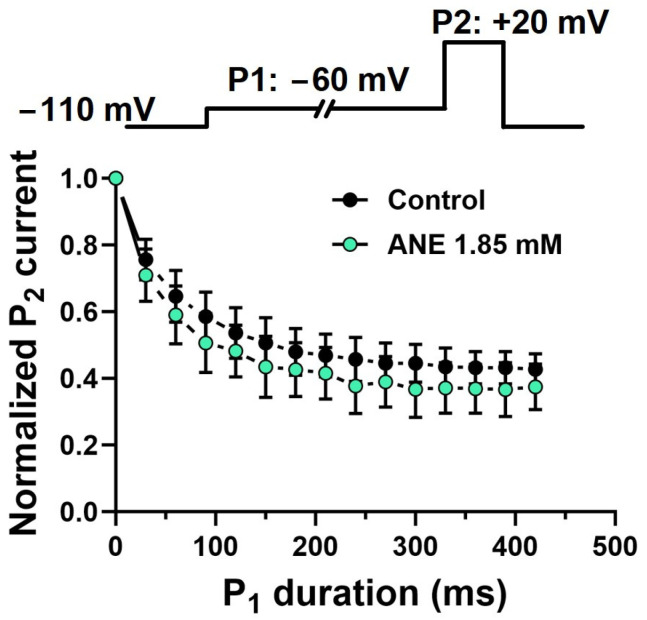
Anethole’s lack of interaction with pre-open states of voltage-gated sodium channels. Averaged fractional current peaks recorded during P2 using the voltage-clamp protocol shown on the top panel. All recordings in this figure were obtained with voltage steps from a holding potential of −110 mV. The effects of ANE on the holding currents are negligible. The computed data from experiments in the absence and in the presence of ANE at 1.85 mM did not differ significantly (ANOVA, n = 6 neurons, *p* > 0.05).

We evaluated the effect of ANE on the pre-open states of the VGSCs that give rise to I_Na+_ in DRG neurons. In these experiments, we used two voltage pulses, P1 and P2, from a holding potential of −110 mV. Pulse P1 consisted of a time-variable conditioning period at −60 mV, and pulse P2 was a fixed +20 mV, 20-ms pulse. The I_Na+_ elicited by pulse P2 after any P1 in the series was normalized with respect to the P2 currents when P1 lasted 0 ms, the first in the series (Figure 5). ANE did not have its I_Na+_ blocking properties enhanced throughout the time series by P1 conditioning pulses, unlike in the control experiments (absence of ANE).

### 2.5. Anethole Shifts the Steady-State Voltage Dependence of Na^+^ Conductance Inactivation

**Figure 6:**
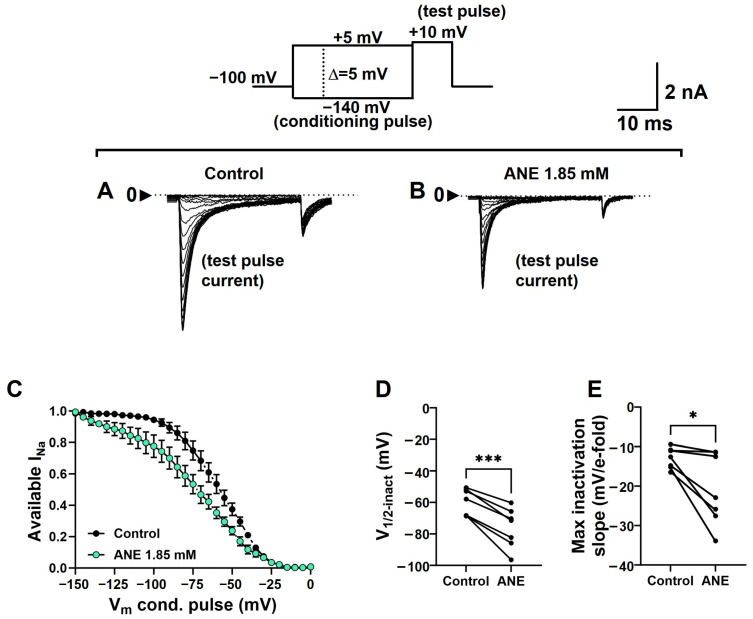
Anethole changes the voltage dependence and sensitivity of I_Na+_ inactivation. (**A**) Typical families of I_Na+_ recorded at +10 mV after 100 ms conditioning periods at voltages ranging from −140 to +5 mV (upper panel) in control conditions (absence of ANE), and (**B**) in the presence of ANE at 1.85 mM concentration. (**C**) Normalized and averaged available I_Na+_ in the absence (control) and in the presence of ANE at a 1.85 mM concentration. Data from individual cells were plotted as individual curves and fitted using Boltzmann’s equation V_1/2_-inact (**D**) and Max inactivation slope (**E**) parameters. * and *** denote *p* < 0.05 and *p* < 0.001 from paired *t* tests (n = 7 neurons), respectively.

To assess the effects of ANE on the I_Na+_ inactivation process, we used a protocol consisting of 100-ms conditioning pre-pulses ranging from −140 to +5 mV, in steps of 5 mV, immediately followed by a 20-ms test pulse to +10 mV. The holding potential in these experiments was −110 mV, and the series of pulses was run at 0.2 Hz to avoid the accumulation of slow I_Na+_ inactivation. I_Na+_ recorded during +10 mV pulses from each cell during this voltage-clamp protocol was normalized by its maximal value (typically when the pre-pulse was −140 mV) and plotted against the respective conditioning pre-pulse voltage. We plotted averaged data to highlight the shift in the voltage dependence in the presence of ANE (Figure 6A–C). We fitted individual curves with Boltzmann’s formalism (Equation (4), see methods) for the extraction of the voltage dependence (V_1/2_-inact) and voltage sensitivity (Max inactivation slope) of the inactivation process. The shift in the I_Na+_ inactivation curves induced by ANE was statistically significant (n = 7, paired *t* test, *p* = 0.0004) and the average curve displacement in the voltage axis induced by ANE was of −16.3 ± 2.33 mV (Figure 6D). ANE also decreased the voltage sensitivity of the I_Na+_ inactivation process (n = 7, paired *t* test, *p* = 0.0205), on average, to 69.1 ± 0.08% of its control values when altered by ANE (Figure 6E).

**Figure 7:**
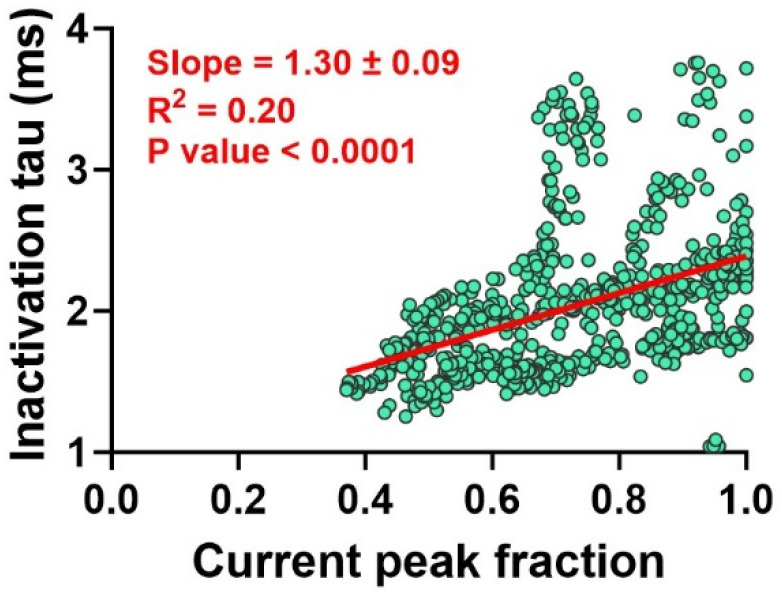
Correlation between I_Na+_ peak fraction and inactivation kinetics. The more intense is the blockade by ANE, the faster the inactivation takes place. The red straight line plotted is the best linear correlation fit to the data (green circles). This correlation shows a significant slope of 1.3 ± 0.09 and R^2^ of 0.20 (n = 773 from 80 neurons, *p* < 0.0001, simple linear regression).

Moreover, we also found a significant correlation between the presence of ANE, quantified by an I_Na+_ fractional peak blockade (shown as an unblocked current peak fraction in Figure 7), and the kinetics of the I_Na+_ inactivation process, shown as inactivation tau. Our analysis shows a slope of 1.3 ± 0.09, which is significantly different from zero (n = 773 from 80 neurons, *p* < 0.0001, simple linear regression, Figure 7), and a correlation coefficient if R^2^ = 0.20.

## 3. Discussion

Our new data show the efficacy of ANE as a VGSC blocker with different affinities to the resting states as compared to the fast-inactivated states of the channels. We determined the effects of ANE on VGSCs naturally expressed in DRG neurons using whole-cell patch-clamp recordings. The blockade induced by ANE on these currents is fully reversible and strongly concentration-dependent, as seen in the Hill slope of ~16. These data show that ANE may modulate cell excitability with similar potency to other terpenes and terpenoids of similarly small chemical structures, such as estragole [9], eugenol [22,23,24,25,26], and carvacrol [27,28]. Furthermore, our data show that ANE blocked I_Na+_ through a mechanism that is like that of lidocaine (a well-known local anesthetic), a blockade that includes binding to the resting and inactive states of the VGSCs [29]. We conclude that ANE is an effective VGSC blocker that possesses the advantageous property of stronger affinity to the channels when these are inactivated, consistent with events of high firing frequency in neurons, as in neurological diseases such as depression, stress, epilepsy, rapid-onset dystonia Parkinsonism, and absence seizures [30,31,32,33,34,35].

VGSCs are essential for the generation and propagation of action potentials, which are an important part of the neuronal information package in the nervous system. These channels are associated with neuronal excitability overall, hence the control of synapse strength throughout the nervous system. In other words, VGSCs are essential for many functions of the nervous system, such as the connection of the body periphery to the central nervous system and inter-organ coordination; skeletal, heart, and smooth muscle contractions; and other functions [36,37,38,39]. Due to the ubiquitous relevance of VGSCs to neuronal physiology, diligent investigation of the effects of any new drug on these channels is necessary.

Dorsal root ganglion neurons express distinct sodium channel isoforms, including Nav1.1, Nav1.6, Nav1.7, Nav1.8, and Nav1.9; each of these isoforms possesses different electrophysiological properties [40,41,42]. Among these channels expressed in DRG neurons, Nav1.7, Nav1.8, and Nav 1.9 are channels preferentially expressed in these cells, and they are associated with mechanisms related to neuropathic and inflammatory pain [29,43,44,45]. Studying natively expressed VGSCs instead of heterologously expressed ones has the advantage of providing the physiologically relevant context of these channels. Ion channels are molecular entities that are thought to be part of macromolecular complexes in the cell membrane. These supramolecular assemblies may vary according to a variety of cell parameters that are still not fully determined [46,47,48,49,50]. The comprehensive concept of drug binding sites may include regions formed by moieties from different proteins in those molecular complexes. However, the direct electrophysiological readout of the interaction between drugs and ion channels may not evidence the existence of such physiological molecular complexes. Therefore, we propose that studies of ANE on VGSCs natively expressed in DRG neurons are optimal since the channels are in their physiological environment and with their putative partners.

ANE is an organic compound present in many scents, drinks, and foods that are habitually consumed by humans [4]. Controlled tests have addressed the safety and toxicity concerns of ANE in the human body, concluding that the molecule is suitable for drug development [51]. Our data show a rapid I_Na+_ blockade upon adding ANE to the experiment. Similarly, the blockade is quickly removed upon ANE washout (Figure 2D). These data indicate that ANE, an overall lipophilic molecule, does not partition in the hydrophobic phase of the membrane lipid bilayer as part of the VGSCs’ blocking mechanism. We rationalize that such a partition would delay the time courses of blocking and unblocking I_Na+_. Instead, our data propose that ANE possesses a classic drug binding site on VGSCs with a defined affinity. Also interesting is the concentration dependency of the concentration–response curve of ANE as an I_Na+_ blocker. We found the Hill slope to be approximately 16. For comparison, this number is around 1 for the blockade of I_Na+_ with lidocaine [29]. This high slope number for ANE could be due to molecular insolubility, but our approach accounted for that, and we believe this is not the case for the results presented here. For this present work, ANE was prepared as a 1 M solution in pure ethanol, and then, diluted to the desired concentration prior to use. Therefore, the ANE/ethanol molar ratio was maintained throughout the several concentrations of ANE used here. The final concentration of ethanol varied in the concentration–response curve. We tested ethanol alone at the equivalent concentration when ANE was used at 5 mM; this was the maximal concentration of ANE in this study, and consequently the maximal concentration of ethanol was 0.42% (*w*/*v*). This amount of ethanol did not block I_Na+,_ in agreement with others’ work [22,23] (Supplementary Figure S1). Our hypothesis to explain the unexpectedly high Hill slope is that ANE may have multiple binding sites on the channels’ molecules (or molecular complexes) and that these multiple sites enable different levels of binding cooperativity and allosterism [24]. Although fascinating from a mechanistic point of view, with possibilities that could be explored, investigating this aspect of ANE is not within the scope of the present study.

Our detailed analysis of the blockade of I_Na+_ induced by ANE revealed interesting features concerning a possible state-dependent blockade. ANE binds to and blocks VGSCs in their resting states. Per our 100 ms pre-conditioning pulse protocol shown in Figure 6C, this voltage prevents inactivation of the channels in the absence of ANE. Hence, with much longer periods at a holding potential of −110 mV, we assume 100% of the channels are in the resting state. ANE interacts with these resting states with an IC50 of 1.85 mM (Figure 2). Weak depolarization to −60 mV, insufficient to activate Na+ conductance, may drive VGSCs to pre-open states and to their inactive states. Should ANE have a higher affinity for these states than that for the resting states, we would observe increased decay of I_Na+_ as the −60 mV pulse duration increased. Such an effect was not observed in our study, as shown in Figure 5. Next, we found no indication of different affinity for the open states of the channels. The fast inactivation of VGSCs may mask or even prevent an open state blockade by ANE. This problem is elegantly featured in previous works [25,26,27,28]. Our data indicate related acceleration of the I_Na+_ inactivation process, but in that case, we can more safely interpret these data as indicating stronger binding to the inactive states of the VGSCs, as discussed below.

Indeed, our data strongly suggest stronger binding to the inactive states of the VGSCs. First, the frequency-dependent effect shown in Figure 3 indicates that the more we give time for the channels to inactivate over a time series, the more ANE blocks I_Na+_ overall. At 5 Hz stimulation and with 50 ms depolarizing pulses (for a 25% duty cycle), ANE increased its overall blockade by about 40% (from 48% to 71%) when compared with 0.2 Hz stimulation with 50 ms depolarizing pulses (for a 0.1% duty cycle). These numbers suggest that ANE at sub-millimolar concentrations may prevent action potentials from being fired in a neuron in its physiologically relevant environment and experimental conditions (voltage clamp vs. current clamp). As with lidocaine, these blockade properties of ANE are of great advantage in that these drugs, more importantly, block the excitability of neurons only when they are firing at high frequencies, such as during some disease states [30,31,32,33,34,35]. Moreover, the voltage-dependent inactivation curve is markedly displaced to more negative potentials in the presence of ANE, suggesting stronger affinity for the inactive states of VGSCs.

We ran an exploratory experiment using ramp depolarizations, instead of voltage steps. With ramp depolarizations, typically, one has an instantaneous current–voltage curve with two distinct negative peaks. The first, taking place at more negative voltages, is related to the opening of those closed states that can transition to inactivated states if time allows. The second, happening at less negative potentials, relates to open states that transition to inactive states [52,53,54]. Our experiment consisted of two depolarization ramps in the same DRG neuron, before and after ANE at 1.85 mM. Our data show that ANE inhibits more intensively the first component of the ramp current, the one related to the states that transition to inactivated states before an open event, when the ramp is fast enough to activate a higher amplitude of I_Na+_ (2 mV/ms ramp compared to 1 mV/ms ramp) (Supplemental Figure S2).

The main core of our data was collected using voltage-clamp protocols from a holding potential (not conditioning pre-pulses) of −110 mV. The protocols were designed to include all nine families of VGSCs (Nav1.1-1.9) in our recordings. Therefore, in this first publication of ours showing ANE as a VGSC blocker, we do not discriminate which VGSC family ANE preferentially blocks. The necessary work to delineate the blockade of specific families of VGSCs by ANE is in the process of conceptualization and will be included in our next project.

ANE is a low-toxicity molecule that we are reporting here as a VGSC blocker. The molecule is liposoluble; its potential uses in vivo, in animal models, or clinically in animals and humans would be primarily as a topical local anesthetic. An analog and similarly liposoluble molecule, menthol, is used in cough drops that are available over the counter to inhibit the cough reflex in humans through a topical anesthesia effect on the throat surface. Other topical anesthetics used in clinics, such as benzocaine and ropivacaine, are used in formulations ranging from 0.25% to 20% for local tissue concentrations of 0.5–1.6 mM [55,56,57]. Nevertheless, comprehensive studies on the effects of ANE on human VGSCs expressed in DRG neurons are necessary for the translational aspect of this molecule. The above aspects were not within the scope of the present study.

Most importantly, our data presented here show the efficacy of ANE as a VGSC blocker, and these new data must be considered when presenting ANE as a new drug to play a role in experimental therapies for neurodegenerative diseases.

## 4. Materials and Methods

### 4.1. Cell Preparation

Dorsal root ganglion (DRG) neurons from 1 to 3-day-old rats were used as a model of natively expressed VGSCs. Briefly, rats were sacrificed through decapitation, and DRGs were quickly removed. All animals were handled in compliance with the Guide for the Care and Use of Laboratory Animals, 8th edition, by the U.S. National Institutes of Health (Washington DC, National Academies Press (US); 2011. ISBN-13: 978-0-309-15400-0ISBN-10: 0-309-15400-6; https://www.ncbi.nlm.nih.gov/books/NBK54050/ accessed on 13 January 2024). Ganglia were digested through a single trypsin 0.25% treatment in a Ca^2+^, Mg^2+^-free Earle’s balanced salt solution (EBSS) containing (mM) 132.8 NaCl, 5.3 KCl, 1 NaH_2_PO_4_, 5.5 glucose, and 10 HEPES, at pH 7.4, adjusted with NaOH. After 15–20 min of digestion, the ganglia were mechanically reduced with a fire-polished Pasteur pipette in a Ca^2+^, Mg^2+^-free EBSS solution containing 5 U/mL DNAse (type I, Sigma-Aldrich, Burlington, MA, USA), 0.15% trypsin inhibitor (type IS, Sigma-Aldrich), and supplemented with 10% fetal calf serum. After pelleting, the cells were resuspended in Dulbecco´s Modified Eagle´s Medium (DMEM, Sigma-Aldrich) supplemented with 10% fetal calf serum, 100 UI/mL penicillin, and 100 μg/mL streptomycin, and seeded on glass coverslips previously treated with poly-L-lysine. This procedure yields DRG neurons ranging from 13 μm to 35 μm in diameter. In addition, these cells are not distributed in different populations in regard to the TTX sensitivity of their I_Na+_. Cells were kept in a water-jacket incubator with the temperature set at 37 °C and in a 5% CO_2_ atmosphere until just before the experiments, which were carried out during the first 7 days after cell isolation.

### 4.2. Anethole Solutions

ANE (Millipore Sigma, CAS Number: 4180-23-8, Mol. Weight: 148.20 g/mol) was prepared as a 1 M stock solution in pure ethanol. On the day of the experiment, this stock solution was diluted with extracellular solution (see recipe below) to the desired working concentration of ANE. The maximal ANE concentration used in this present study was 5 mM, which equates to 0.42% ethanol or 92 mM ethanol in the working solution. As a reference, tests run with 200 mM ethanol on voltage-activated Na^+^ currents showed that this 2.3-fold higher concentration had no significant effect on the Na^+^ currents [23].

### 4.3. Electrophysiology

Total sodium current (I_Na+_) passing through VGSCs was recorded using voltage clamping with the conventional whole-cell patch-clamp configuration. Patch pipettes were fabricated from borosilicate glass capillaries using a model P-97 micropipette puller (Sutter Instrument, Novato, CA, USA). Patch pipettes were pulled to achieve initial bath resistances averaging 2 MΩ when filled with an intracellular solution containing (in mM) 10 NaCl, 150 CsF, 10 TEA-chloride, 1 ATP, 4.5 MgCl_2_, 9 EGTA, 10 HEPES, and pH 7.3, adjusted with CsOH. CsF and TEA-chloride were used to eliminate K^+^ currents. Cells were bathed during the recordings in an extracellular solution containing (in mM) 82 choline chloride, 50 NaCl, 1.2 MgCl_2_, 1.8 CaCl_2_, 1 CoCl_2_, 4 KCl, 5 glucose, 10 HEPES, and pH 7.4, adjusted with NaOH. CoCl_2_ was used to eliminate voltage-activated Ca^2+^ currents from the recordings. Typical access resistance values were below 3 MΩ. Command voltage waveforms were generated on a computer by Clampex 10 software (Molecular Devices, San Jose, CA, USA), and a digital-to-analog converter (model 1322, Molecular Devices) delivered the analog waveform signal to the Axopatch 200B patch-clamp amplifier (Molecular Devices), which held the pipette voltage. The current recordings were low-pass filtered at 5 kHz by a built-in Bessel filter in the amplifier, sampled at 25–50 kHz, and at 16 bits by an analog-to-digital converter for the final recording on a hard disk for further analysis. The cell membrane capacitance was canceled, and access resistance was routinely compensated (85% for both prediction and compensation; lag set to 10 μs). The P/-4 protocol [58] was used to eliminate uncompensated capacitive currents and leak currents from the recorded data. However, this technique was not used when probing the use-dependent block by ANE to avoid underestimation if the overall recovery from the inactivation processes. We used a −110 mV holding potential as a standard procedure to always remove channels from inactivation when not pulsing. In addition, when possible, we pulsed at 0.2 Hz throughout the time series of the protocol for the same reason. All recordings were performed between 20 °C and 23 °C. During the experiments, the recording chamber was continuously perfused with bath solution to avoid unstirred layers.

### 4.4. Data Analysis and Graphs

Scientific data were processed, analyzed, and plotted using Clampfit 10 (Molecular Devices, Foster City, CA, USA), GraphPad Prism 10 (GraphPad Software, LLC, La Jolla, CA, USA), Origin (OriginLab, Northampton, MA, USA), and Microsoft Excel (Microsoft, Redmond, WA, USA). Averaged data (symbols plotted in graphs) were computed from at least six independent experiments or otherwise noted. The vertical bars in the graphs are the standard error of the mean (SEM).

The equations for the data fittings mentioned in the Results section are as follows:

Dose–response curves were fitted using Hill’s formalism:

(1) $$Normalized\;unblocked\;I_{Na+}=\frac{{\left[ANE\right]}^{nH}}{{\left[ANE\right]}^{nH}+{{IC}_{50}}^{nH}}$$

where the Normalized unblocked I_Na+_ is the remaining I_Na+_ after blockade by *ANE*, *IC_50_* is the concentration of ANE that blocks 50% of the I_Na+_, and nH is the Hill’s coefficient.

Current–voltage relationships were transformed into Na^+^ conductance (G) versus voltage (V) curves (G-V curves) by using Ohm’s law:

(2) $$G_{Na+}=\frac{I_{peak}}{V_{m}-V_{r}}$$

where G_Na+_ is the Na^+^ conductance at a certain membrane potential V_m_, and V_r_ is the reversal potential for Na^+^ in the experimental conditions.

G-V curves were fitted using the following equation:

(3) $$Normalized\;{Na}^{+}conductance=\frac{1}{1+e^{\left(\frac{V_{1/2-act}-V_{m}}{Max\;slope-act}\right)}}$$

where Normalized Na^+^ conductance is the fractional conductance activated at a given membrane potential V_m_, V_1/2-act_ is the membrane potential for half-maximal Na^+^ conductance activation (the midpoint), and Max slope-act is the voltage sensitivity of the activation by the voltage process.

Na^+^ currents inactivated by voltage curves (inactivation curves) were fitted using the following equation:

(4) $$Available\;I_{Na+}=1-\frac{1}{1+e^{\left(\frac{V_{1/2-inact}-V_{m}p-p}{Max\;slope-inact}\right)}}$$

where Available I_Na+_ is the Na^+^ current after the conditioning pre-pulse voltage period V_m_p−p, V_1/2-inact_ is the V_m_p−p that inactivates half of I_Na_, and Max slope-inact is the voltage sensitivity of the inactivation by the voltage process.

Na^+^ current inactivation kinetics were fitted using the following exponential function:

(5) $$I_{Na+}=\left(A\times 1-e^{-t/\tau }\right)+y_{0}$$

where I_Na+_ is the Na^+^ current at a given moment t, A is the amplitude of the exponential, τ is the time constant, and y_0_ is an adjusting factor for persistent currents.

### 4.5. Statistical Analysis

Data from individual cells were treated individually, including for fitting purposes. Pooled fitting parameters from distinct groups, e.g., ANE vs. control (its absence), were compared using a paired t test to detect consistent changes in the parameters that relate to the drugs. Levels of significance were * *p* < 0.05, ** *p* < 0.01, *** *p* < 0.001, and **** *p* < 0.0001.

## 5. Conclusions

In conclusion, the present work investigated, in detail, the blocking properties of ANE on VGSCs natively expressed in sensory neurons. ANE possesses a stronger binding affinity to the inactive states of VGSCs. The data presented here are thought to contribute to developing a comprehensive understanding of ANE as a molecule that is biologically active in neural tissue. In addition, our data may also pave the way to further studies that aim to improve ANE’s binding specificity and safety through molecule-smart and oriented modifications. Further, our results provide a possible mechanism for the anesthetic action of ANE and contribute to the development of new compounds that can be used in pain treatments.

## Data Availability

The data presented in this study are available from the corresponding author upon reasonable request.

## Supplementary Materials

The following supporting information can be downloaded at: https://www.mdpi.com/article/10.3390/ijms25021034/s1.
