# Supplementary material for: State-Dependent Blockade of Dorsal Root Ganglion Voltage-Gated Na+ Channels by Anethole
Source: Int J Mol Sci. 2024 Jan 14;25(2):1034. doi: 10.3390/ijms25021034 (PMC10816912; doi:10.3390/ijms25021034)
Supplement: Supplementary file 1 [file ijms-25-01034-s001.zip › ijms-2786713-supplementary.pdf]

Article

State-Dependent Blockade of Dorsal Root Ganglion Voltage-Gated Na$^+$ Channels by Anethole

Luiz Moreira-Junior [1], Jose Henrique Leal-Cardoso [2], Antonio Carlos Cassola [3] and Joao Luis Carvalho-de-Souza [1,3,*]

[1] Department of Anesthesiology, University of Arizona, Tucson, AZ 85724, USA; luizmoreira@arizona.edu

[2] Superior Institute of Biomedical Sciences, State University of Ceará, Campus of Itaperi, Fortaleza 607402, CE, Brazil; lealcard@gmail.com

[3] Department of Physiology and Biophysics, Biomedical Sciences Institute, University of Sao Paulo, São Paulo 05508, SP, Brazil; cassola@icb.usp.br

* Correspondence: jcds@arizona.edu

SUPPLEMENTARY MATERIALS

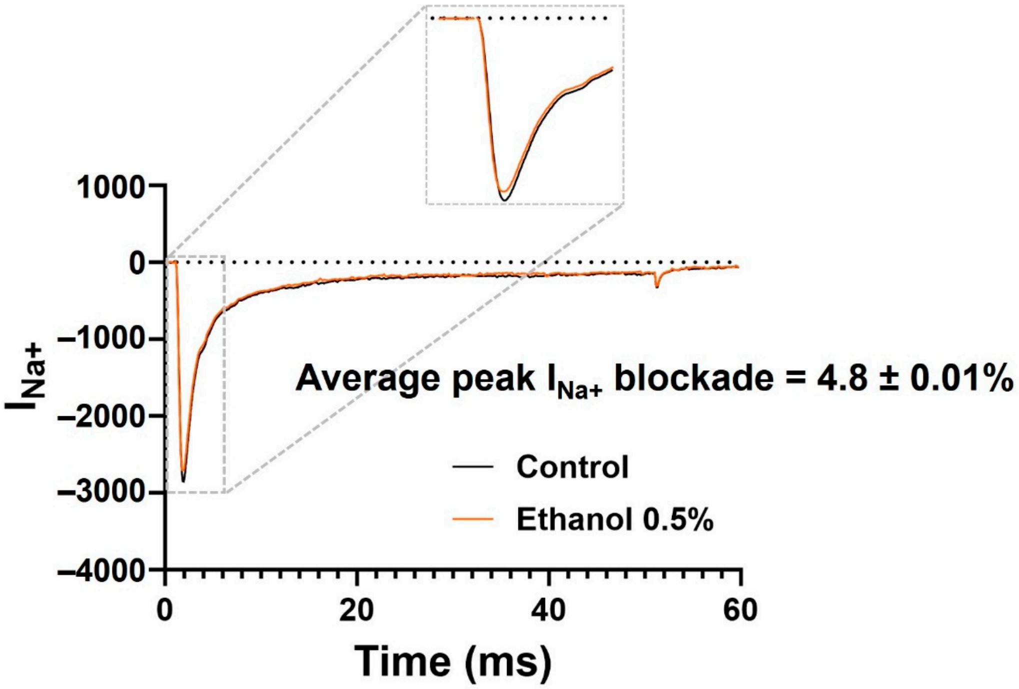

Figure S1. Effects of 0.5% ethanol on INa+ recorded on DRG neurons. This 0.5% ethanol concentration was used with 5 mM ANE and proportionally with smaller concentrations.

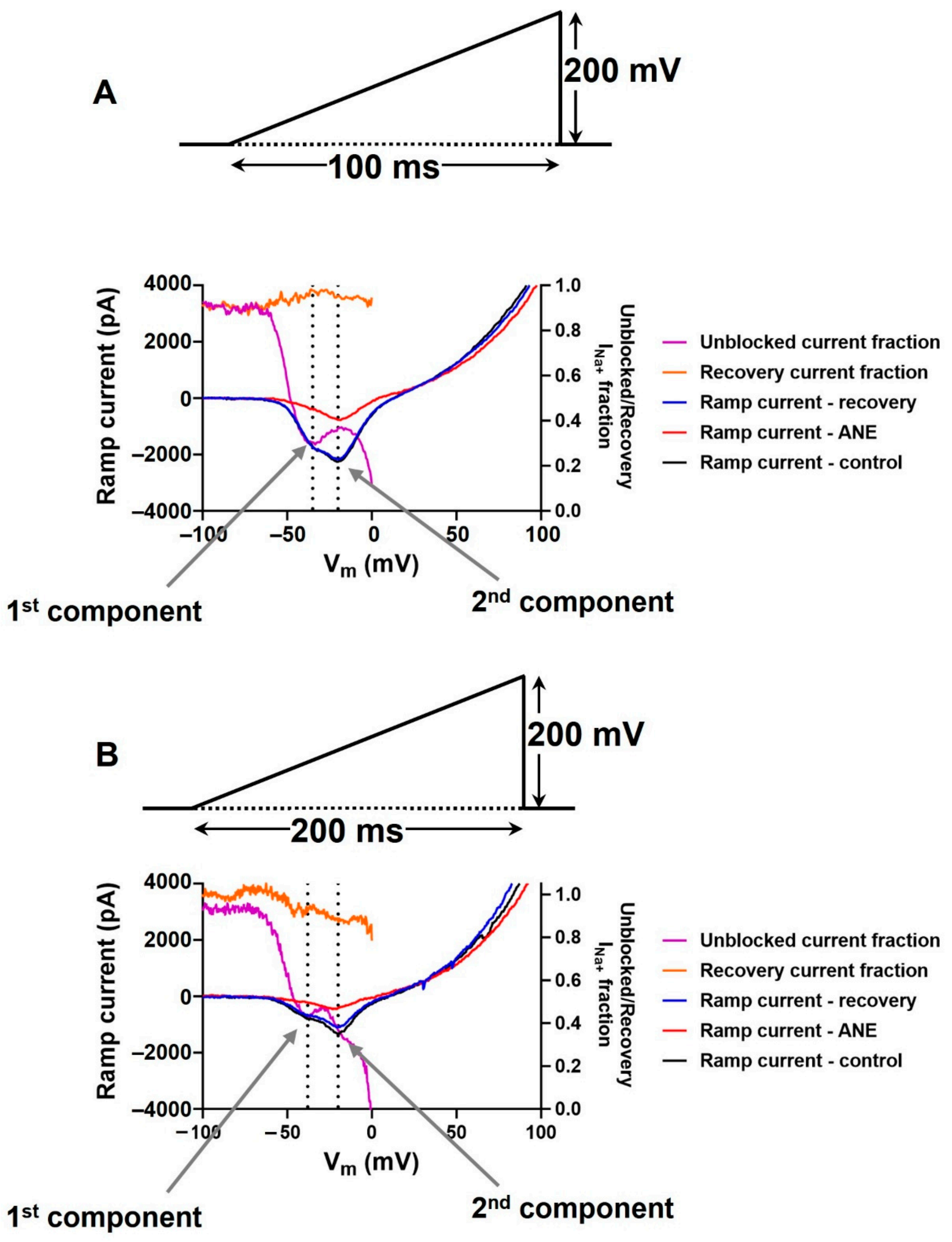

Figure S2: Preliminary ramp INa+ at different mV/ms rates before, during and after the application of ANE at 1.85 mM.
